# Hydration and Diffusion of H^+^, Li^+^, Na^+^, Cs^+^ Ions in Cation-Exchange Membranes Based on Polyethylene- and Sulfonated-Grafted Polystyrene Studied by NMR Technique and Ionic Conductivity Measurements

**DOI:** 10.3390/membranes10100272

**Published:** 2020-10-01

**Authors:** Vitaliy I. Volkov, Alexander V. Chernyak, Daniil V. Golubenko, Vladimir A. Tverskoy, Georgiy A. Lochin, Ervena S. Odjigaeva, Andrey B. Yaroslavtsev

**Affiliations:** 1 Institute of Problems of Chemical Physics RAS, 142432 Chernogolovka, Russia; chernyak@icp.ac.ru (A.V.C.); lochin.g@yandex.ru (G.A.L.); odzhigaeva.ervena@yandex.ru (E.S.O.); 2 Scientific Center in Chernogolovka RAS, 142432 Chernogolovka, Russia; 3 Kurnakov Institute of General and Inorganic Chemistry RAS, 119991 Moscow, Russia; xpman2009@yandex.ru (D.V.G.); yaroslav@igic.ras.ru (A.B.Y.); 4 Lomonosov Institute of Fine Chemical Technologies, MIREA–Russian Technological University, 119571 Moscow, Russia; tverskoy@mitht.ru; 5 Faculty of Chemistry, Lomonosov Moscow State University, 119991 Moscow, Russia

**Keywords:** sulfonic cation-exchange membrane, hydration number, pulsed field gradient NMR, diffusion coefficient, ionic conductivity

## Abstract

The main particularities of sulfonate groups hydration, water molecule and alkaline metal cation translation mobility as well as ionic conductivity were revealed by NMR and impedance spectroscopy techniques. Cation-exchange membranes MSC based on cross-linked sulfonated polystyrene (PS) grafted on polyethylene with ion-exchange capacity of 2.5 mg-eq/g were investigated. Alkaline metal cation hydration numbers (h) calculated from temperature dependences of ^1^H chemical shift of water molecule for membranes equilibrated with water vapor at RH = 95% are 5, 6, and 4 for Li^+^, Na^+^, and Cs^+^ ions, respectively. These values are close to h for equimolar aqueous salt solutions. Water molecules and counter ions Li^+^, Na^+^, and Cs^+^ diffusion coefficients were measured by pulsed field gradient NMR on the ^1^H, ^7^Li, ^23^Na, and ^133^Cs nuclei. For membranes as well as for aqueous chloride solutions, cation diffusion coefficients increased in the following sequence: Li^+^ < Na^+^ < Cs^+^. Cation and water molecule diffusion activation energies in temperature range from 20 °C to 80 °C were close to each other (about 20 kJ/mol). The cation conductivity of MSC membranes is in the same sequence, Li^+^ < Na^+^ < Cs^+^ << H^+^. The conductivity values calculated from the NMR diffusion coefficients with the use of the Nernst–Einstein equation are essentially higher than experimentally determined coefficients. The reason for this discrepancy is the heterogeneity of membrane pore and channel system. Ionic conductivity is limited by cation transfer in narrow channels, whereas the diffusion coefficient characterizes ion mobility in wide pores first of all.

## 1. Introduction

Ion-exchange membranes are widely applied for separation processes, particularly targeted ion extraction from aqueous solutions. Electrochemical technology development and new material generation require the investigation of the ionic transfer mechanism [1,2,3]. Ion-exchange membrane conductivity is determined by water uptake, charge group nature and polymeric matrix structure [4,5]. The hydration degree of membranes is mainly determined by water coordination to cations. Nuclear magnetic resonance (NMR) techniques provide unique information on the composition of the hydrate complexes and membrane ionic channel framework. The first results of cation hydration in sulfonic cation resins based on sulfonated polystyrene Dowex 50 W and in aqueous acid and salt solutions, as model systems, were published at the end of the 1960s and the beginning of the 1970s just after commercialization of NMR spectrometers [6,7,8,9,10]. It was shown that water molecules in the first hydration sphere of cation are polarized, destroying the hydrogen bond network. Therefore, the water ^1^H NMR line is shifted in a high magnetic field. In the case of the hydrogen ion form, the H^+^ counter ion forms additional hydrogen bonds caused by the low field ^1^H NMR line shift. Hydration cations H(H_2_O)_h_^+^ (h is hydration number) are generated. It was shown that at low humidity in sulfonic cation-exchange resin CU-2 [11], Nafion [12,13,14] and MF-4SC [15,16] membranes acidic protons form hydroxonium ions H_5_O_2_^+^. The hydration of other cations—for instance, alkaline metal cations—has not been sufficiently studied [15], in spite of it being very important for revelation of membrane ion selectivity mechanism.

For membrane transfer processes investigation, the cation diffusion is especially interesting, as it is directly connected with ionic conductivity. The mobility of water molecules is also very important, since they are directly involved in cations transport [15,17,18,19,20]. This leads to a significant dependence of the membrane ionic conductivity on humidity. The diffusion coefficients of water in the ion-exchange membranes decrease by several orders of magnitude with the decreasing of a water uptake λ (λ is amount of water molecules per sulfonate group) if λ ≤ h [12,15,16,17,18]. Proton conductivity in the Nafion membrane is changed in similar manner [12,21,22,23]. Pulsed field gradient NMR (PFG NMR) gives a unique opportunity to measure self-diffusion coefficients directly. Till now PFG NMR experiments in membranes were carried out on ^1^H nuclei of water molecules and hydrated H^+^ cations and only their average self-diffusion coefficient was estimated [12,15,16,17,18]. In order to determine cation and water molecule self-diffusion coefficients separately, the NMR measurement on cation nuclei like ^7^Li, ^23^Na, ^133^Cs is necessary. It is not simple because of low NMR sensitivity of these nuclei. Therefore, the self-diffusion of Li^+^, Na^+^, and Cs^+^ in ion exchangers is low investigated by PFG NMR technique. Grafted ion-exchange membranes on the basis of manufacturing polymer films are very promising. Cation-exchange membranes based on polyethylene (PE) grafted with sulfonated polystyrene (SPS), which we will call “MSC membranes” in accordance with the originally proposed name [24], have shown excellent transport performance and a high potential for power generation systems such as fuel cells and reverse electrodialysis plants. [24] Hydrophilic segments SPS are formed in hydrophobic PE matrix. Recent years of research have shown that the transport properties of this kind of membranes are not worse than a Nafion membrane [24,25,26]. The possibility of Li^+^, Na^+^, and Cs^+^ cation diffusion coefficients measurements in grafted ion-exchange membranes by pulsed field gradient NMR on the ^7^Li, ^23^Na, and ^133^Cs nuclei was shown for the first time in our previous investigation [27]. Therefore, there is an opportunity to compare the ionic diffusion and conductivity measurements correctly.

The main objective of this work is to characterize hydration, diffusion and conductivity of alkaline metal Li^+^, Na^+^, and Cs^+^ cations in polyethylene membranes with grafted sulfonated polystyrene. To explain the regularities of the transfer processes, a comparative study of the cation hydration and mobility in aqueous solutions of lithium, sodium, and cesium chlorides, as in model systems, has been carried out. The interconnection between cation hydration and diffusion are discussed.

## 2. Materials and Methods

### 2.1. MSC Membrane Synthesis, Ion-Exchange Capacity and Humidity Measurements, and Sample Preparations

Sulfonated cation-exchange membranes were obtained by post-radiation grafting polymerization of styrene on a pre-oxidized low-density PE film with a thickness of 20 µm followed by sulfonation of grafted PS with 96% sulfuric acid at temperature 98 °C, as described in references [24,27]. To generate peroxides in a PE film, the latter was irradiated in air at a ^60^Co γ-radiation source with an irradiation dose power of 5.2 Gy/s to absorbed irradiation doses of 0.05 and 0.1 MGy. Post-radiation chemical-grafting polymerization was carried out in a styrene/methanol mixture (1/1 by volume) containing iron(II) sulfate as a peroxide reducing agent. The degree of PS grafting (Δ*p*) was calculated from the weight gain of the film.

(1) $$\Delta p=\left[\frac{\left(m_{1}–m_{0}\right)}{m_{0}}\right]\cdot 100\%$$

where *m*_1_ is the mass of polystyrene grafted sample and *m*_0_ is the mass of the sample (PE film) before grafting.

The measurement of the ion-exchange capacity (IEC, mEq/g) was carried out according to the State Standard GOST 20255.1-89 and GOST 20255.2-89. A sample of the dry cation exchange membrane in proton form was weighed and placed in a dry conical flask with an NaOH solution. The flask was sealed with a stopper and stirred for several hours. The NaOH solution was then poured into a dry beaker, and the sample was titrated with a standard HCl solution. The calculation of IEC was carried out according to the standard procedure. A membrane with IEC of 2.5 mg-eq/g was studied in this work.

To determine the water uptake, the membranes were balanced with saturated salt solutions, after which the membrane was weighed. The membrane was then dried at 80 °C to constant weight in a vacuum created by a foreline pump. The water uptake of the ion exchange membrane was calculated by mass loss, which was characterized by the amount water molecules per sulfonate group (λ)

(2) $$\lambda =\frac{m_{H_{2}O}}{m_{dry}\cdot M\left(H_{2}O\right)\cdot IEC}$$

where $m_{H_{2}O}$ and m_dry_ are the mass of water in the membrane and mass of the dry membrane and M (H_2_O) = 18 g/mol is the molar mass of water [12,14,27]. The λ values at different relative humidity RH for Li^+^, Na^+^, Cs^+^ ionic forms MSC membrane are given in Table 1.

To prepare the sample for NMR measurements, the membrane was cut into small strips, weighed, and placed in desiccators containing saturated solutions of salts of MgCl_2_ (RH = 32%), NaBr (RH = 58%), NaCl (RH = 78%), and Na_2_CO_3_ (RH = 95%). Membrane samples were kept in desiccators until a constant weight. The samples were placed in a standard 5 mm sample tubes, which was hermetically sealed. The measurements were carried out in the 20 °C to 80 °C temperature range.

To standardize membranes in the H^+^ form, the initial samples were kept for 24 h in a 1 M HCl solution and then washed with distilled water. To transfer the membrane to the Li^+^ form, the sample was kept in a 1 M solution of lithium hydroxide (with a tenfold excess) for 24 h, after which it was thoroughly washed with distilled water. The completeness of the conversion to the Li^+^ form was determined by ^1^H NMR spectra.

To transfer the membrane to the Na^+^ form, the sample in the Li^+^ form was kept in a 1-M solution of sodium chloride (with a tenfold excess) for 24 h, after which it was thoroughly washed with distilled water. The completeness of the conversion to the Na^+^ form was determined by ^7^Li NMR spectra.

To transfer the membrane to the Cs^+^ form, the sample in the Na^+^ form was kept for 24 h in a 1-M solution of cesium sulfate or cesium chloride (with a tenfold excess) and thoroughly washed with distilled water. The completeness of the conversion to the Cs^+^ form was determined by ^23^Na NMR spectra.

### 2.2. Experimental Technique

#### 2.2.1. NMR Spectroscopy, Diffusion Coefficient Measurement

High-resolution ^1^H, ^7^Li, ^23^Na, and ^133^Cs NMR spectra were recorded with the use of Bruker Avance III-500 and Avance III-400 WB Fourier transform NMR spectrometers (Bruker, Ettlingen, Germany).

**Figure 1:**
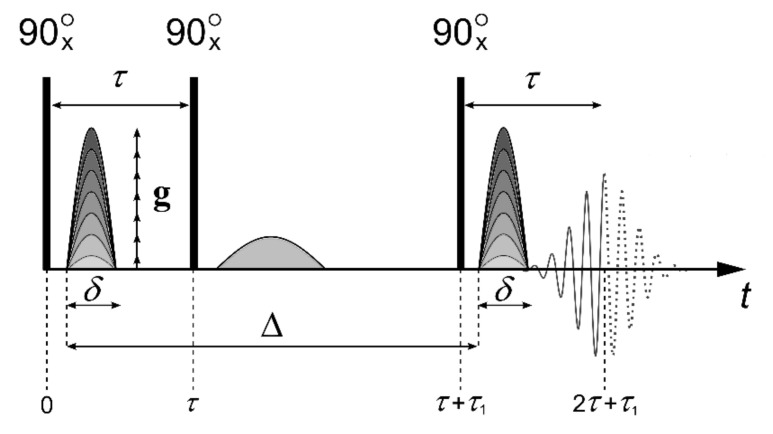
Stimulated echo pulse sequence with the magnetic field gradient pulses.

Diffusion coefficient (DC) measurement of water molecules and Li^+^, Na^+^, and Cs^+^ cations were carried out using a NMR Fourier transform Bruker Avance III-400 WB spectrometer equipped with a pulsed magnetic field gradient probe. The maximum value of the pulsed gradient amplitude was 30 T/m. Diffusion coefficients were measured at frequencies of 400.22, 155.51, 105.84, and 52.48 MHz for ^1^H, ^7^Li, ^23^Na, and ^133^Cs nuclei, respectively. The stimulated echo pulse sequence was used to measure diffusion coefficients (Figure 1).

Here, τ is the time interval between the first and second radio frequency (RF) pulses, *τ*_1_ is the time interval between the second and the third pulses, ∆ is the interval between the gradient pulses, *δ* is duration of the equivalent rectangular magnetic field gradient pulses, and g is the amplitude of the magnetic field gradient pulse [28].

For the molecules undergoing unhindered isotropic Brownian motion, the evolution of the spin echo signal is described by the following equation:

(3) $$A\left(2\tau ,\tau_{1},g\right)=A\left(2\tau ,\tau_{1},0\right)\exp\left(-\gamma^{2}g^{2}\delta^{2}t_{d}D\right)$$

where γ is gyromagnetic ratio, *t_d_* = Δ − *δ*/3 is the diffusion time; D is the diffusion coefficient; and τ, τ_1_, and *g* are parameters explained in Figure 1.

Accordingly, $A\left(2\tau ,\tau_{1},0\right)$ is expressed by the following equation:

$$A\left(2\tau ,\tau_{1},0\right)=\frac{A\left(0\right)}{2\exp\left(-\frac{2\tau }{T_{2}}-\frac{\tau_{1}}{T_{1}}\right)}$$

where *A*(0) is the signal intensity after the first RF pulse (Figure 1). *T*_1_ and *T*_2_ are the spin-lattice and spin-spin relaxation times, respectively. While measuring the echo signal evolution, τ and τ_1_are fixed, and only the dependence *A* of as a function of *g* is analyzed, which is called the diffusion decay.

In the case of non-exponential decays, the experimental curves

$$A\left(g\right)=\frac{A\left(2\tau ,\tau_{1},g\right)}{A\left(2\tau ,\tau_{1},0\right)}$$

are usually deconvoluted in several exponential components, which are described by Equation (3). For the multiphase system consisting of *m* phases in the case of slow (compare to *t_d_*) molecular exchange between the phases,

(4) $$A(g)=\sum_{i=1}^{m}p_{i}^{’}\exp(-\gamma^{2}g^{2}\delta^{2}t_{d}D_{i})$$

where *D_i_* is the diffusion coefficient of *i*-th component and

$${p^{\prime }}_{i}=p_{i}\exp\left(-\frac{2\tau }{T_{2i}}-\frac{\tau_{1}}{T_{1i}}\right)/\sum_{i=1}^{m}p_{i}\exp\left(-\frac{2\tau }{T_{2i}}-\frac{\tau_{1}}{T_{1i}}\right)$$

$$\sum_{i=1}^{m}p_{i}=1$$

Here, *p_i_* is the relative amount of the nuclei belong to the molecules characterized by the diffusion coefficient *D_i_*. The value *p_i_* is called the population of *i*-th phase. For the long T_1_ and T_2_ values, it is usually assumed that *p_i_* ≈ *p^′^_i_*. The details of the experimental curve decomposition in several exponential diffusion decays were described previously [27,28].

In the literature, the term “self-diffusion coefficient” is often used for the diffusion coefficient measured by the pulsed field gradient method. We are applying the term “diffusion” because this term is usual among the membrane scientists.

#### 2.2.2. Ionic Conductivity Measurement

Ion conductivity was measured using an Elins Z1500J (Chernogolovka, Russia) impedance meter (frequency range 1 kHz–1.5 MHz) on symmetric carbon/membrane/carbon cells with an active surface area S of 0.5 cm^2^. The conductivity value *σ* (S∙cm^–1^) was calculated from the resistance *R* found from the impedance hodographs from the cutoff on the axis of active resistances and the geometric dimensions of the membrane according to the Equation (5) The Binder MKF 115 constant climate chamber was used to set the required humidity and temperature during measurement.

(5) $$\sigma =\frac{l}{S\cdot R}$$

where *l* is the membrane thickness in cm, *S* is membrane area in cm^2^.

A typical impedance hodograph is shown in Figure S1. We considered the electrical equivalent circuit describing this system in Golubenko, D., Karavanova, Y., Yaroslavtsev’s article [29]. With an increase in the current frequency, the polarizing contribution of diffusion layers decreases, accompanied by a decrease in the real and imaginary parts of the complex resistance. At high frequencies, the imaginary part of the impedance decreases to zero, while the real part of the impedance is equivalent to the membrane’s ohmic resistance. We found the ohmic resistance directly by extrapolating the hodographs to the active resistance axis in this work.

## 3. Results and Discussion

### 3.1. H, ^7^Li, ^23^Na, ^133^Cs NMR Spectroscopy. Hydration Numbers

^1^H, ^7^Li, ^23^Na, and ^133^Cs NMR spectra of MSC membranes are represented by narrow lines that belong to water molecules, protons, and Li^+^, Na^+^, and Cs^+^ cations in appropriate ionic forms of the membrane (Figures S2 and S3). A low line width (no more than 1 kHz) indicates high mobility of water and ions in the membranes. ^1^H NMR spectrum of MSC membrane in hydrogen form is represented by two singlet lines (Figure S2).

The line with the highest intensity belongs to the protons of water molecules and hydrated H^+^ cations (in the acidic ionic form of the membrane). The low intensity signal at 2–3 ppm very likely belongs to mobile fragments of polyethylene matrix [27].

**Figure 2:**
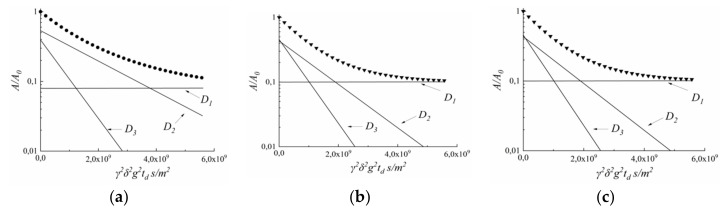
Spin echo attenuation (diffusion decay) of water molecule ^1^H nuclei in Li^+^ ionic form (**a**), Na^+^ ionic form (**b**) and Cs^+^ ionic form (**c**) of MSC membrane; RH = 95%, T = 293K. Dots are experimental curves; straight lines are decomposition on D_1_, D_2_, D_3_ components from Equation (4). Components of diffusion coefficients D_1_, D_2_, and D_3_ and relative parts p_1_, p_2_, and p_3_ are the next. For Li^+^ ionic form, MSC D_1_ = (2.4 ± 0.5) 10^−13^ m^2^/s, D_2_ = (5.0 ± 0.5)·10^−10^ m^2^/s, D_3_ = (1.3 ± 0.5)∙10^−9^ m^2^/s and (0.08 ± 0.01), (0.53 ± 0.05), (0.39 ± 0.05), correspondingly. For Na^+^ ionic form, MSC (4.5 ± 0.5)·10^−13^ m^2^/s, (7.8 ± 1)·10^−10^ m^2^/s, (1.5 ± 0.5)·10^−9^ m^2^/s and (0.1 ± 0.015), (0.44 ± 0.05), (0.46 ± 0.05), correspondingly. For Cs^+^ ionic form MSC D_1_ = (3.4 ± 0.3) 10^−13^ m^2^/s, D_2_ = (1.1 ± 0.2)·10^−9^ m^2^/s, D_3_ = (1.7 ± 0.2)·10^−9^ m^2^/s, and (0.13 ± 0.1), (0.49 ± 0.05), (0.38 ± 0.04), correspondingly.

Water molecules in MSC membranes are not uniformly distributed. Spin echo attenuation (diffusion decay) of ^1^H nuclei is a non-linear shape it is a sum of three exponential components approximating by Equation (4). The ^1^H diffusion decays in Li^+^, Na^+^, and Cs^+^ ionic forms of MSC membrane at RH = 95% are shown in Figure 2.

Component with the least diffusion coefficient D_1_ (2.4–4.5) × 10^−13^ m^2^/s belongs to signal at 2–3 ppm (Figure S2). It may be proposed that this signal is due to the low molecular weight of polyethylene fragments arising during γ-irradiation.

Diffusion coefficients D_2_ and D_3_ (10^−10^–10^−9^ m^2^/s) are typical of water molecules (or hydrated cation H^+^ in acid ionic form of MSC membrane). The existence of water molecules with different translation mobility denotes on membrane heterogeneity.

The diffusion coefficient D_3_ practically does not depend on the type of cation and is (1.3–1.7) × 10^−9^ m^2^/s, which is close to the diffusion coefficient of bulk water (2.4 × 10^−9^ m^2^/s). These water molecules are rather far from cations are probably belong to the so-called “uncharged solution” in the wide membrane pores. We suppose that translation mobility of water molecules coordinated by cation is partially characterized by diffusion coefficient D_2_. Thermogravimetry techniques enable us to calculate the integral water uptake λ only. The number of water molecules connected with cation λ_s_ may be calculated as the product of its relative part, and λ: λ_s_ = p_2_λ/(p_2_ + p_3_). Namely, λ_s_ values are using for cation hydration number h calculation.

Let us mention some features of low field ^1^H signal in H^+^ form of MSC membrane. The NMR line width increases, and the line center position is shifted to the low field with humidity and temperature decrease. This chemical shift is larger compare to bulk water, which indicates proton hydration with the formation of H(H_2_O)^+^_h_ [27].

In contrast to the hydrogen form, in salt forms of MSC membranes, the ^1^H NMR line is shifted to strong fields. This is due to the fact that water molecules located in the first coordination sphere of cations do not act as acceptors of hydrogen bonds and turn out to be less polarized compared to bulk water molecules forming a hydrogen bond network. Consequently, the hydrogen bond network is partially destroyed [15].

**Figure 3:**
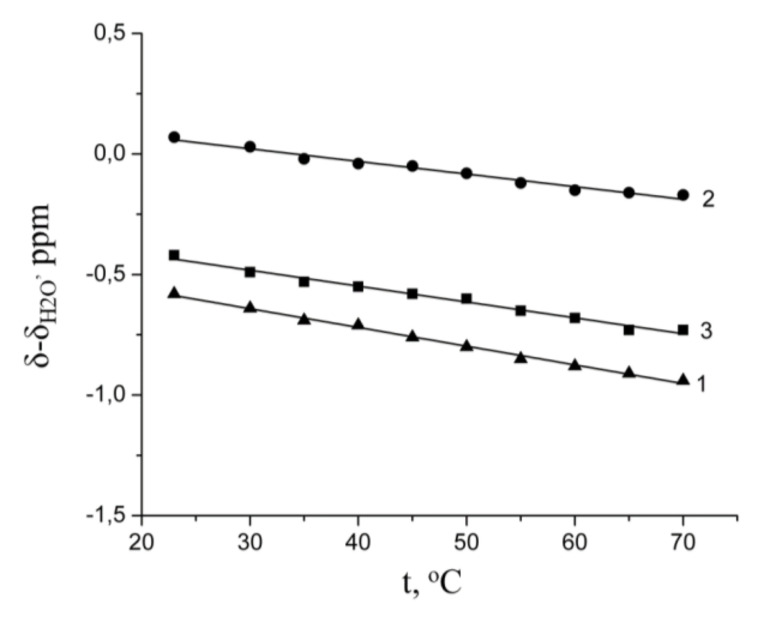
Chemical shift of water molecule ^1^H nuclear temperature dependences in Li^+^(1), Na^+^(2) Cs^+^(3) ionic forms at RH = 95%.

The fast exchange takes place between water molecules in the first hydrated spheres of cation and other water molecules. Therefore, the observed ^1^H signal is singlet of which chemical shift *δ* may be approximate as [7,10]

(6) $$\delta =\frac{\delta_{h}\cdot h}{\lambda_{s}}+\frac{\delta_{H_{2}O}\cdot (\lambda_{s}-h)}{\lambda_{s}}$$

where *h* is the hydration number, *λ_s_* is the water molecule amount per cation (sulfonated group), which we have calculated above from Figure 2. Keeping in mind that *δ_h_* is temperature-independent because of water molecules in the first cation hydrated sphere polarization, we have the next equation for chemical shift temperature dependence:

(7) $$\frac{d\delta }{dt}=\frac{\left(\lambda_{s}-h\right)}{\lambda_{s}\frac{d\delta_{H_{2}O}}{dt}}$$

which is presented by a straight line (Figure 3)

From the ^1^H chemical shift, the temperature dependences the hydration numbers *h* of Li^+^, Na^+^, and Cs^+^ cations in MSC membrane were calculated for the first time using Equation (8) [7,10].

(8) $$h=\lambda_{s}\left[1-\frac{\frac{d\delta }{dt}}{\frac{d\delta_{H_{2}O}}{dt}}\right]$$

where *dδ*/*dt* is a chemical shift temperature dependence of membrane water, b is the slope of *dδ*/*dt* temperature dependence, which are 0.0068, 0.0052, 0.0066 for Li^+^, Na^+^, and Cs^+^ ionic forms, correspondingly; *dδ_H2O_*/*dt* is the a chemical shift temperature dependence of bulk water, b is 0.01 [8].

**Figure 4:**
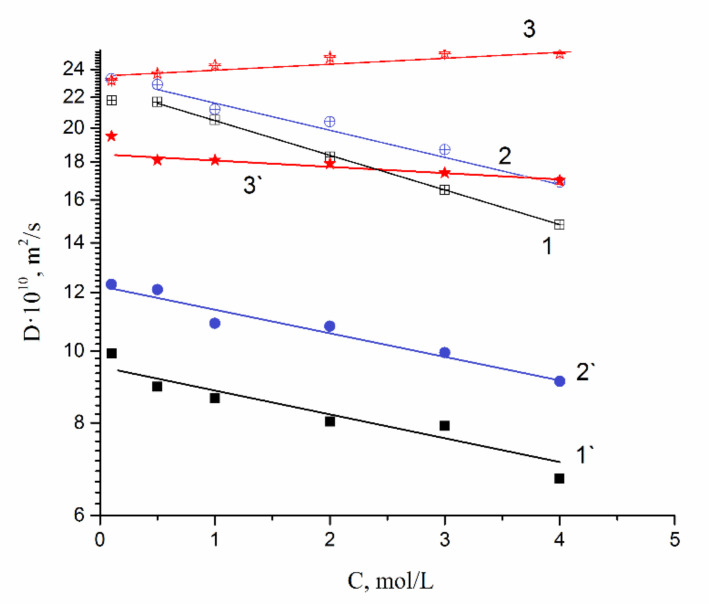
Water molecule and cation diffusion coefficient concentration dependences in lithium, sodium, cesium chloride aqueous solutions. 1–H_2_O in LiCl, 2–H_2_O in NaCl, 3–H_2_O in CsCl. 1′–Li^+^ in LiCl, 2′–Na^+^ in NaCl, 3′–Cs^+^ in CsCl.

Hydration numbers h of Li^+^, Na^+^, and Cs^+^ are shown in Table 2. The hydration numbers for same cations in equimolar aqueous salt chloride solutions and membrane water uptakes are also shown for comparison. The h values of Li^+^, Na^+^, and Cs^+^ in MSC membranes at high humidity is practically equal to salt solution ones. The crystallography radii and Stokes–Einstein hydrodynamic ion radii are also presented in Table 2. We have calculated hydrodynamic ion radii from the Stokes–Einstein equation on the basis of ionic diffusion coefficient in chloride aqueous solution. Ion diffusion coefficient concentration dependences (Figure 4) were approximated to an infinite dilute concentration.

### 3.2. Diffusion of Li^+^, Na^+^, and Cs^+^ Cations and Ionic Conductivity

#### 3.2.1. Diffusion of Li^+^, Na^+^, and Cs^+^ Cations in MSC Membrane

**Figure 5:**
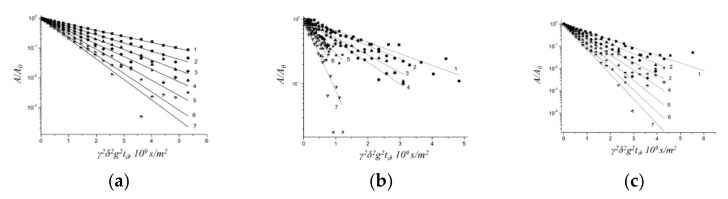
Diffusion decays of ^7^Li (**a**), ^23^Na (**b**), ^133^Cs (**c**) nuclei NMR signals in appropriate ionic form of MSC membrane at RH = 95% and different temperatures 1–20 °C, 2–30 °C, 3–40 °C, 4–50 °C, 5–60 °C, 6–70 °C, 7–80 °C.

Spin echo attenuation (diffusion decay) of ^7^Li^+^, ^23^Na^+^, and ^133^Cs^+^ is exponential in salt ionic form of MSC membrane. Diffusion decay is well approximated by Equation (3) (Figure 5).

**Figure 6:**
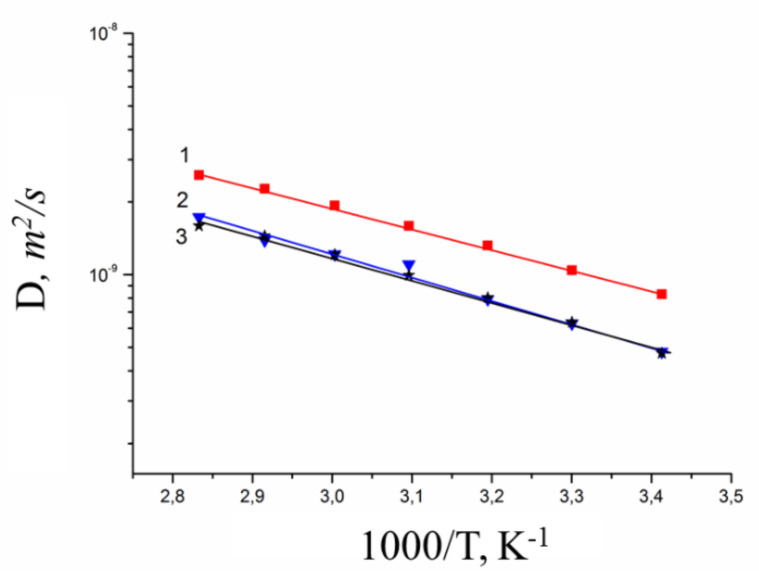
Temperature dependences of Cs+, Na+, and Li+ diffusion coefficients in appropriate ionic form of MSC membrane at RH = 95%: 1–Cs+ ionic form, Ea = 18.1 kJ/mol; 2–Na+ ionic form, Ea = 16.5 kJ/mol; 3–Li^+^ ionic form, E_a_ = 17.6 kJ/mol.

Diffusion coefficient temperature dependences are shown in Figure 6. These dependences are linearized in the coordinates of the Arrhenius equation,

(9) $$D=D_{0}\cdot e^{-\frac{E_{a}}{R\cdot T}}$$

where *D*_0_ is temperature independent, *R* is gas constant, *T* is absolute temperature, *E_a_* is a diffusion activation energy.

Cation diffusion coefficients increase in a sequence Li^+^ ≈ Na^+^ < Cs^+^. This row is the same for cation diffusion coefficients of chloride aqueous solutions (Figure 4). Cation diffusion activation energies are about 16–18 kJ/mol.

#### 3.2.2. Ionic Conductivity of MSC Membrane

**Figure 7:**
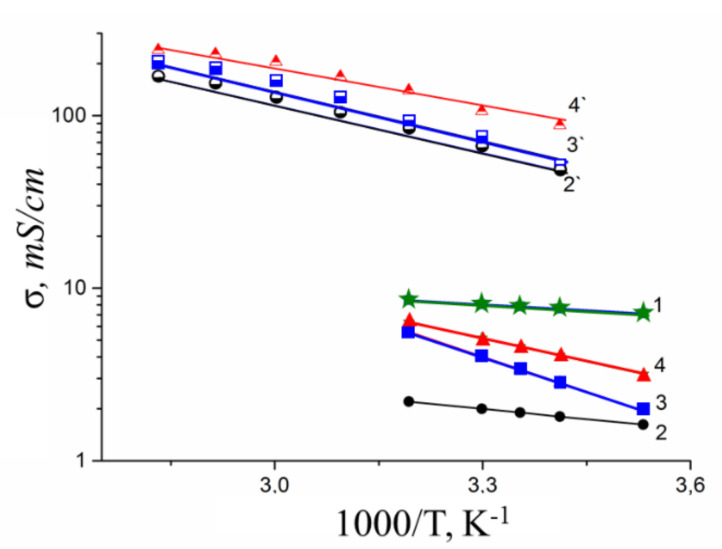
Temperature dependences of experimental σ_exp_ (1–4) and calculated σ_calc_ (2′–4′) ionic conductivities in H^+^ (1), Li^+^ (2) and (2‘), Na^+^ (3) and (3′), and Cs^+^ (4) and (4′) ionic forms of MSC membrane at RH = 95%.

Temperature dependences of ionic conductivity of different ionic forms of MSC membranes are shown in Figure 7.

The conductivity values at different humidity and activation energies calculated from Arrhenius equation are listed in Table 3.

Ionic conductivity of investigated membranes increases in the sequence Li^+^ < Na^+^ < Cs^+^ << H^+^. It should be noted that the diffusion coefficients of lithium, sodium, and cesium cations in MSC and in aqueous solutions change in the same sequence. Ionic conductivities (σ_calc_) of MSC membranes were calculated using Nernst–Einstein Equation (10).

(10) $$\sigma_{calc}=\frac{N\cdot D\cdot e^{2}}{k\cdot T}$$

where *N* is a number of charge carrier in cm^3^; *D* is diffusion coefficient, m^2^/s; *e* is electron charge, 1.9 × 10^−19^ C; *k*-Boltzmann constant, 1.38 × 10^−23^ J/K; *T* is absolute temperature, *K*.

As shown in Figure 7, the calculated and experimental conductivity curve are similar. Conductivity activation energies of alkaline metal cations are close to each other. However, the calculated conductivity values are one or two orders of magnitude more in comparison with experimental ones. This difference seems natural. Ionic transport in membranes is realized through the system of channels and pores, which size is depended on polymeric matrix nature and hydration degree. So, for example, in accordance with the well-known Gierke model, at high humidity, the size of pores in perfluorinated membranes of the Nafion type is 4–5 nm. At the same time, the diameter of the channels connecting them is much smaller and is about 2 nm [32,33]. It is well known that ionic conductivity is limited by the transport of ions in the narrow channels. They are usually called the “bottle neck” [34] Ionic transfer in narrow channels namely limits a membrane ionic conductivity [35]. It may be supposed that the diffusion coefficient measured by NMR in the first turn is due to high mobility ions localized in wide pores [27]. Indeed, simple estimation shows that the volume of water in the pores of the Nafion membranes is about an order of magnitude higher than in the channels connecting them. The concentration of cations in membrane’s pore solution increases with decreasing of surrounding relative humidity. The fixed –SO_3_^−^ groups are much less hydrated compared to cations. From this point of view, it will be sensible to investigate the chemical shifts and diffusion coefficients of water and cation in aqueous chloride solutions depending on concentration as in a simple model system.

#### 3.2.3. Li^+^, Na^+^, and Cs^+^ Hydration and Diffusion in Chloride Aqueous Solutions

**Figure 8:**
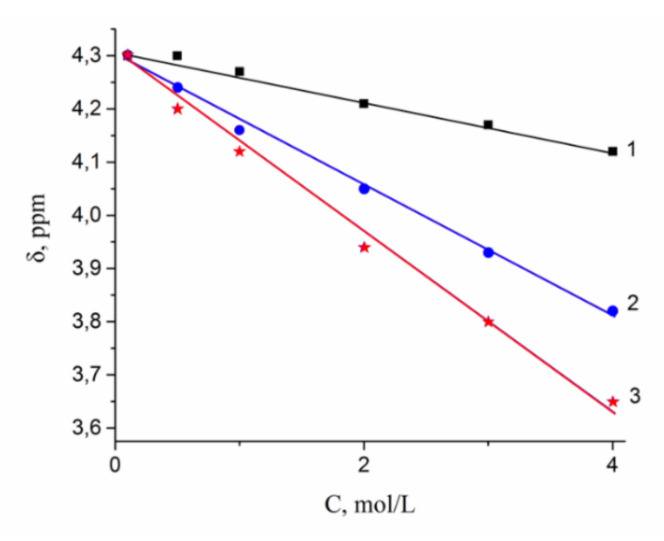
1H chemical shift dependences on concentration of LiCl (1), NaCl (2), and CsCl (3) aqueous solutions.

A dependence of ^1^H water molecule chemical shift on solution concentration is shown in Figure 8. Proton signal shifts to the high field with chloride concentration increasing. This fact is explained by destroying of hydrogen bonds between water molecules [7,8,15,36]. This phenomenon is stronger for the CsCl solution, since Cs^+^ possesses low polarizing properties (due to the large ion size), which causes the hydrogen bond system to be destroyed.

As a result, water molecule translational mobility should increase with increasing concentration, which is observed experimentally (curve 3 in Figure 8).

The water molecules and cation diffusion coefficient increase with alkaline metal atomic mass increase. The mobility of cations increases in the sequence Li^+^, Na^+^, and Cs^+^ due to an increase in the water mobility and due to a decrease in the effective radius of the hydrated cation. Both of these phenomena are associated with decreasing the hydration energy in the same order. It is important to note that, in contrast to the data obtained for membranes, the diffusion coefficients of dilute solutions found using NMR practically coincide with the diffusion coefficients described in the literature on the base of ion conductivity data [37]. This emphasizes that the discrepancy between the data for membranes is determined by the difference in the nature of the ion mobility founded by NMR and conductometry. NMR data characterize the ion mobility in the pores of the membrane, but ionic conductivity is limited by the transfer in narrower channels.

It should be mentioned that Li^+^, Na^+^ and water diffusion coefficients are reduced greater compared to Cs^+^ ion for which water diffusion coefficient even increases with an increase in electrolyte concentration (curves 1′, 2′;1, 2 (Figure 4) and curves 3′, 3 (Figure 4)). The water hydrogen bond network is destroying to a greater extent with increasing in Cs^+^ concentration. As contrasted to Cs^+^, hydration energy of Li^+^ and Na^+^ ions is more. Therefore, the mobility of water molecules connected with these cations drops and water diffusion coefficient is reduced with the rise in salt concentration.

Now, the reason for the decrease in the diffusion coefficients of cations with an increase in the concentration of solutions should be considered. To understand this, it is worth noting that water molecules are bound by both cations and anions, forming a more or less ordered environment of water molecules around them. To implement the cation transfer, it must destroy its coordination environment and form a new one. Obviously, the formation of a new environment is difficult when the ordered environment of another ion is located nearby. The higher the concentration of the solution, the higher the fraction of relatively tightly bound water molecules surrounded by cations, the more difficult the process of ion transfer is. In aqueous solutions, this is expressed in a clearly pronounced tendency toward a decrease in the activity coefficient of ions with an increase in their concentration.

**Figure 9:**
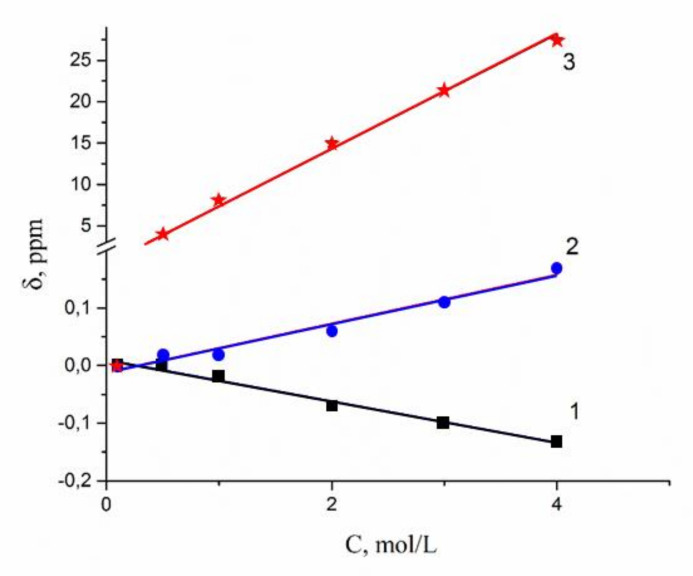
^7^Li (1), ^23^Na (2), and ^133^Cs (3) nuclear NMR chemical shift concentration dependences in lithium, sodium, cesium chloride aqueous solutions.

In Figure 9, ^7^Li, ^23^Na, and ^133^Cs nuclei chemical shift concentration dependences are shown. This dependence is stronger for ^133^Cs. Chemical shift increases (compare to ^7^Li, ^23^Na), while CsCl concentration is varied from 1 mol/L to 4 mol/L (curve 3, Figure 9).

The chemical shift of these nuclei is determined by nuclear quadrupole moment interaction with electric field gradient, created by nearest hydrated water molecules. Lithium and sodium cations form of rather stable and symmetric complexes. Therefore, an electric field symmetry and a nuclear chemical shift are changed slightly with concentration variation (curves 1,2 Figure 9). Cesium cation surrounding is not stable because of a weak bond Cs^+^ with water molecules. With an increase of CsCl solution concentration, the symmetry of the surrounding Cs^+^ decreases; this is a reason for the increase of the ^133^Cs chemical shift (curve 3, Figure 9).

## 4. Conclusions

The comparison of hydration, diffusion, and ionic conductivity in MSC membranes and chloride aqueous solutions shows that in high humidity membrane (RH = 95%) hydration numbers of Li^+^, Na^+^, and Cs^+^ is closed to those in dilute aqueous solutions. Cation diffusion coefficients and ionic conductivity increase in a sequence of Li^+^ < Na^+^ < Cs^+^. The conductivity values calculated from the NMR diffusion coefficients using the Nernst–Einstein equation are essentially higher than experimentally determined ones. These results are discussed on the basis of ^1^H, ^7^Li, ^23^Na, and ^133^Cs chemical shift and water molecule and Li^+^, Na^+^, and Cs^+^ cation diffusion coefficient experimental dependences in chloride aqueous solutions. In contrast to the data obtained for membranes, the diffusion coefficients of dilute solutions measured by NMR practically coincide with the diffusion coefficients described in the literature, calculated from ion conductivity data. This emphasizes that the discrepancy between the experimental and calculated conductivities for membranes is determined by the difference in the nature of the ionic mobilities determined by NMR and conductometry. NMR characterizes the ion mobility in the wide pores of the membrane, but ionic conductivity is limited by the ion transfer in narrow channels.

## Figures and Tables

**Table 1:** Water uptake λ at different relative humidity RH for Li^+^, Na^+^, and Cs^+^ ionic forms of MSC membrane.

RH, %	λ, [H_2_O]/[SO_3_Li], Li^+^ Ionic Form	λ, [H_2_O]/[SO_3_Na], Na^+^ Ionic Form	λ, [H_2_O]/[SO_3_Cs], Cs^+^ Ionic Form
12	0.6	0.8	0.0
33	1.4	1.1	1.4
58	3.3	3.1	3.4
75	5.7	3.9	3.7
84	8.1	6.2	6.6
98	23.5	20.9	16.1

**Table 2:** Crystallography radii, Stokes–Einstein hydrodynamic ion radii, and hydration numbers (h) of Li^+^, Na^+^, and Cs^+^ cations in appropriate MSC membrane ionic forms at RH = 95% and in equimolar aqueous salt chloride solutions.

Cation	Li^+^	Na^+^	Cs^+^
Crystallography ionic radius, Å [30]	0.69	1.02	1.67
Stokes-Einstein hydrodynamic ionic radius, Å [31]	2.38	1.84	1.19
Stokes-Einstein hydrodynamic radius, estimated from ionic diffusion coefficient in chloride aqueous solution at infinite dilute concentration	2.7	2.2	1.5
Total water uptake of membrane (λ)	24	21	16
Water amount per membrane sulfonate group (λ_s_)	13.8	10.3	8.1
Hydration number of cations (h) in membrane	4.1 ± 1	5.0 ± 1	3.1 ± 1
Hydration number of cations (h) in aqueous solution [7,8]	4	4.6	3.9

**Table 3:** Experimental values of ionic conductivity at 25 °C and conductivity activation energies of H^+^, Li^+^, Na^+^, and Cs^+^ cations in MSC membrane with different humidity. IEC = 2.5 mg–eq/g.

RH, %	95		75		58		32	
Ionic Form	Ea, kJ∙mol^−1^	σ _exp_ mS∙cm^−1^	Ea, kJ∙mol^−1^	σ _exp_ mS∙cm^−1^	Ea, kJ∙mol^−1^	σ _exp_ mS∙cm^−1^	Ea, kJ∙mol^−1^	σ _exp_ mS∙cm^−1^
H	4.3	7.8	11	6	12	3	23	0.6
Li	7.5	1.9	30	0.5	39	0.2	52	0.008
Na	26	3.4	31	0.7	40	0.2	68	0.01
Cs	17	4.6	32	0.8	37	0.3	60	0.02

## Supplementary Materials

The following are available online at https://www.mdpi.com/2077-0375/10/10/272/s1, Figure S1: (A) EEC for a conductor with predominantly ionic conduction Figure S1 A - EEC for a conductor with predominantly ionic conduction; (B) Typical Nyquist plot of MSC-membrane. Here is Cs-form at 75% RH; Figure S2: ^1^H NMR spectrum in H^+^ ionic form of MSC membrane at RH = 95% and T = 293K; Figure S3: NMR spectra of ^7^Li (a), ^23^Na (b) and ^133^Cs (c) nuclei in appropriate ionic form of MSC membrane at RH = 95%.
