# Supplementary material for: Hydration and Diffusion of H+, Li+, Na+, Cs+ Ions in Cation-Exchange Membranes Based on Polyethylene- and Sulfonated-Grafted Polystyrene Studied by NMR Technique and Ionic Conductivity Measurements
Source: Membranes (Basel). 2020 Oct 1;10(10):272. doi: 10.3390/membranes10100272 (PMC7601212; doi:10.3390/membranes10100272)
Supplement: Supplementary file 1 [file membranes-10-00272-s001.pdf]

membranes

Supplementary Materials: Hydration and Diffusion of H^+, Li^+, Na^+, Cs^+ Ions in Cation-Exchange Membranes Based on Polyethylene- and Sulfonated-Grafted Polystyrene Studied by NMR Technique and Ionic Conductivity Measurements

Vitaliy I. Volkov [1,2,*], Alexander V. Chernyak [1,2], Daniil V. Golubenko [3], Vladimir A. Tverskoy [4], Georgiy A. Lochin [1,5], Ervena S. Odjigaeva [1,5], and Andrey B. Yaroslavtsev [3]

1 Institute of Problems of Chemical Physics RAS, Chernogolovka 142432, Russia; chernyak@icp.ac.ru (A.V.C.); lochin.g@yandex.ru (G.A.L.); odzhigaeva.ervena@yandex.ru (E.S.O.)
2 Scientific Center in Chernogolovka RAS, Chernogolovka 142432, Russia
3 Kurnakov Institute of General and Inorganic Chemistry RAS, Moscow 119991, Russia; xpman2009@yandex.ru (D.V.G.); yaroslav@igic.ras.ru (A.B.Y.)
4 Lomonosov Institute of Fine Chemical Technologies, MIREA–Russian Technological University, Moscow 119571, Russia; tverskoy@mitht.ru
5 Faculty of Chemistry, Lomonosov Moscow State University, Moscow 119991, Russia
* Correspondence: vitwolf@mail.ru or vitwolf@icp.ac.ru

Received: 12 August 2020; Accepted: 28 September 2020; Published: 1 October 2020

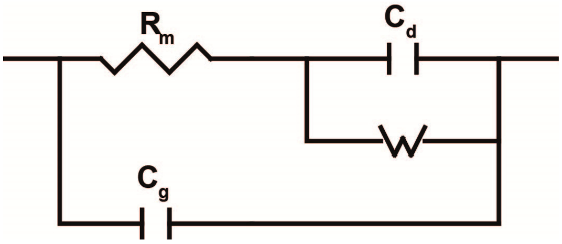

A

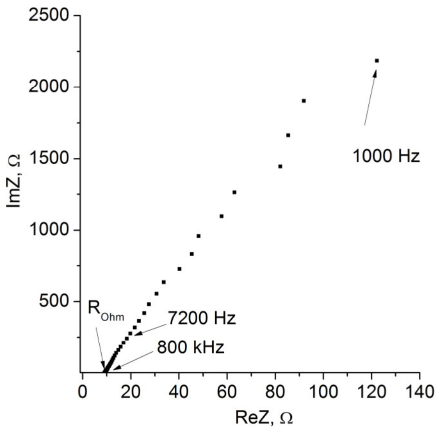

B

Figure S1. (**A**) EEC for a conductor with predominantly ionic conduction; (B) Typical Nyquist plot of MSC-membrane. Here is Cs-form at 75% RH.

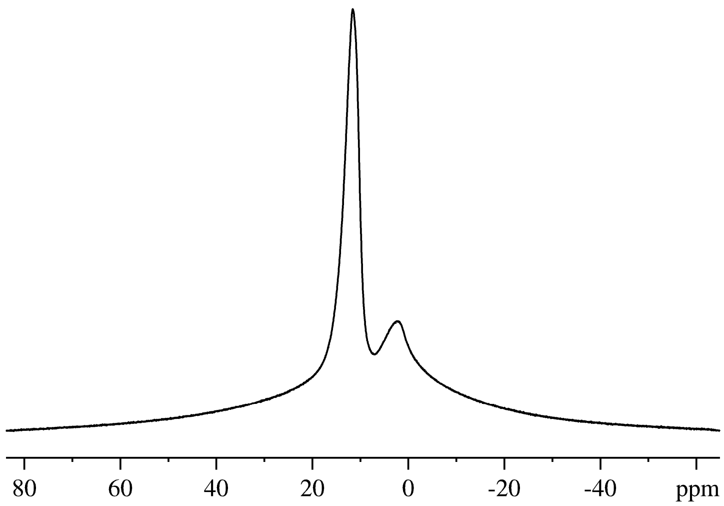

Figure S2. ^1H NMR spectrum in H^+ ionic form of MSC membrane at RH=95% and T = 293K

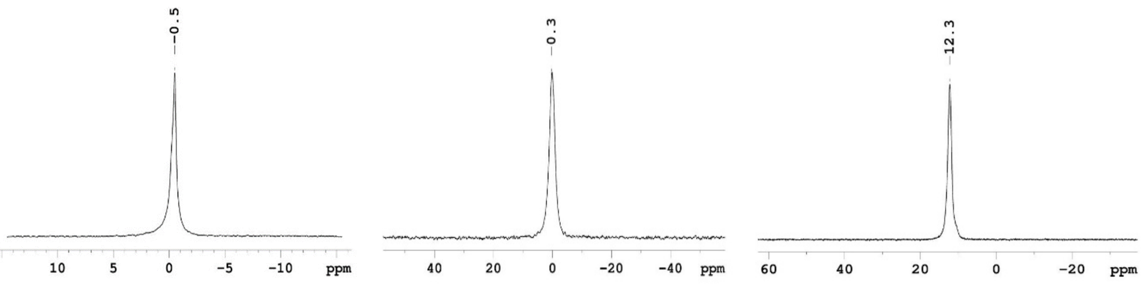

a b c

Figure 3. NMR spectra of ^7Li (**a**), ^{23}Na (**b**) and ^{133}Cs (c) nuclei in appropriate ionic form of MSC membrane at RH = 95%.

© 2020 by the authors. Licensee MDPI, Basel, Switzerland. This article is an open access article distributed under the terms and conditions of the Creative Commons Attribution (CC BY) license (http://creativecommons.org/licenses/by/4.0/).
